# Charge-Controlled Energy Optimization of the Reconstruction of Semiconductor Surfaces: *sp*^3^–*sp*^2^ Transformation of Stoichiometric GaN(0001) Surface to (4 × 4) Pattern

**DOI:** 10.3390/ma17112614

**Published:** 2024-05-29

**Authors:** Pawel Strak, Wolfram Miller, Stanislaw Krukowski

**Affiliations:** 1 Institute of High Pressure Physics, Polish Academy of Sciences, Sokolowska 29/37, 01-142 Warsaw, Poland; stach@unipress.waw.pl; 2 Leibniz Institute for Crystal Growth (IKZ), Max-Born-Str. 2, D-12489 Berlin, Germany; wolfram.miller@ikz-berlin.de

**Keywords:** charge balance, surface reconstruction, density functional theory, gallium nitride

## Abstract

It was demonstrated by ab initio calculations that energy optimization in the reconstruction of semiconductor surfaces is controlled by the global charge balance. The charge control was discovered during simulations of the influence of heavy doping in the GaN bulk, which changes *sp*^3^ to *sp*^2^ ratio in the reconstruction of stoichiometric GaN(0001), i.e., a Ga-polar surface. Thus, the reconstruction is not limited to the charge in the surface only; it can be affected by the charge in the bulk. The discovered new reconstruction of the GaN(0001) surface is (4 × 4), which is different from the previously reported (2 × 1) pattern. The undoped GaN reconstruction is surface charge controlled; accordingly, (3/8) top-layer Ga atoms remain in a standard position with *sp*^3^ hybridized bonding, while the remaining (5/8) top-layer Ga atoms are shifted into the plane of N atoms with *sp*^2^ hybridized bonding. The change in the charge balance caused by doping in the bulk leads to a change or disappearance of the reconstruction pattern.

## 1. Introduction

Despite their importance, semiconductor surfaces are relatively less known than the other properties of semiconductors. On the other hand, these surfaces are important from the point of view of the technology, and they are also an interesting subject of basic research. Their scientific advancement is reflected in the language used for the description of their properties. The properties of metal surfaces are described using up-to-date quantum mechanical methods including ab initio calculations. Thus, in a relatively simple approach, semiconductor surfaces were and still are described within broken-bond models [1]. This was not changed by the recent application of DFT calculations to some selected semiconductor surfaces as these investigations were mostly limited to the configurational aspect only.

Primarily, the semiconductor surfaces differ from the metallic surfaces by their propensity toward reconstruction. The reconstructed surfaces have their translational symmetry lowered as compared to direct termination surfaces. In some cases, this may occur due to the presence of additional atoms having a specified ordering. In the case of the adsorption of additional species, the number of possible variants is immensely large as any adsorbate can be strongly attached by the bonding to the atoms of the surface. Such reconstructions are, therefore, not suitable for studies to obtain a fundamental understanding of the phenomenon as different chemical components may affect the surface differently, thus obscuring the intrinsic nature of the system.

It is, therefore, preferable to study the stoichiometric surfaces first, which creates an opportunity to establish their basic features more clearly. Nevertheless, the variety of the configurations can be still rich. In the absence of the additionally attached atoms, reconstruction occurs via the modification of the crystallographic structure. The depth of the modification can vary, in the case of the Si(111) surface, the three atomic layers are shifted [2]. In the case of the GaAs(100) surface, this reconstruction involves the creation of As dimers, i.e., a single atomic layer [3]. Another possibility for simple reconstruction is a shift of fraction of atoms into differently bonded positions in a spatially oriented pattern.

Surface reconstructions were investigated by ab initio calculations in many different semiconductors, including GaAs [3,4,5,6], GaN [7,8,9], or CdTe [10]. These studies were limited to the determination of the atomic configuration only. The electronic component was not considered explicitly. This is in disagreement with the other investigation of the surface properties. In recent studies of adsorption, it was demonstrated that electronic contribution plays a decisive role in the process outcome [11]. It was shown that electron contribution changes the adsorption energy of hydrogen at the H-covered GaN(0001) surface by several electronvolts; i.e., it dominates the final result [12,13,14]. Large-scale contributions to the adsorption energy change were identified for the adsorption of ammonia at a H/NH_3_-covered GaN(0001) surface [13,15], nitrogen at a N-covered AlN(0001) surface [16], silicon at a Si-covered SiC(0001) surface [17], or hydrogen at a H-covered GaN(000-1) surface [18]. Therefore, the importance of the electronic component is a universal feature of adsorption at semiconductor surfaces that manifests itself as a jump-like change in the adsorption energy at the critical coverage corresponding to the Fermi level not pinned (free) at the surface. This could be explained using the electron counting rule (ECR) [4] or even more completely using the extended version of the ECR (EECR) [15].

It is, therefore, expected that not only adsorption but also other phenomena, such as reconstruction, may depend on the charge balance and the Fermi level pinning. This was not investigated; surface reconstruction is interpreted exclusively in terms of the simple broken-bond model where the Fermi energy is absent. Therefore, we selected polar Ga-terminated stoichiometric GaN(0001) as a case study for determining the role of charge in the reconstruction. This effect was not discovered in any surface; thus, the properties need careful investigation. On one hand, it is well known that a GaN stoichiometric surface is extremely difficult to obtain by standard methods [19]. Nevertheless, by cleavage at high vacuum in an extremely low-pressure, low-temperature environment, used for quantum condensate experiments, it is possible to obtain such a surface. On the other hand, gallium nitride and its solid solutions with InN and AlN continue to remain in the focal point of the research directed toward the development of new generations of optoelectronic and electronic devices, such as light emitting diodes (LEDs), laser diodes (LDs), and superluminescent emitting diodes (SLEDs) potentially covering the entire visible and a large portion of the UV spectral range [20,21]. Other more sophisticated electro-optical devices could be designed in the future using this or different materials [22]. In parallel, the nitride-based electronic devices designed to be developed also include high-power and high-electron-mobility transistors (HEMTs) [23]. Thus, the nitride-based devices offer an important contribution to the presently emerging new-technology civilization based on carbon-free energy production, distribution, and consumption [24]. These devices are grown on a GaN(0001) surface; therefore, the investigation of this surface has technological importance. Therefore, this surface is a suitable subject for investigation not only from a fundamental point of view but also for applications.

The initial ab initio calculations of the limited size of the slabs used (2 × 2) supercells. Therefore, in the early research, the ab initio determined properties of clean GaN(0001) indicated an absence of any reconstruction [25,26,27,28]. That was later changed by the identification of (2 × 1) [7,8]. Both configurations are dominated by the gallium broken-bond quantum state of large dispersion, located in the upper part of the energy gap. The state is partially filled, pinning the Fermi level at the surface; i.e., the surface is metallic. In the present work, these findings will be verified using large-size slab simulations. The charge analysis will be used to elucidate the basic relationship between the charge and the reconstructions. A natural extension will be the use of bulk doping to determine the connection between the charge in the bulk and the surface reconstruction. The relation between reconstruction and the charge will be established.

The plan of this paper is as follows: In Section 2, we present the simulation method along with the test of the obtained result. In Section 3, the main body of the results is presented, divided into the semi-insulating (SI) and n-type and p-type doped GaN bulk. The simulations contain different slab sizes to remove the influence of the parallel-size slab influence on the stable configurations. The simulations data are supplemented with the charge analysis showing that the selected pattern is compatible with the charge occupation of the upper $\left({sp}^{3}\right)$ and lower $\left({sp}^{2}\right)$ energy surface states. The data are presented together with the band diagram proving identification of the surface states. Subsequently, the doped GaN slabs, both p-type and n-type by the introduction of substitutional Mg and Si atoms, are presented. It is shown that, in the case of n-type doping, the fraction of these two types of hybridized atoms is changed, again in accordance with the global charge balance. Finally, the summary is presented, and conclusions are drawn.

## 2. The Calculation Procedure

Ab initio density functional theory (DFT) calculations were used in the simulations of the properties of the large-size GaN slabs representing the Ga-polar stoichiometric GaN(0001) surface. These calculations employ the Spanish Initiative for Electronic Simulations with the Thousands of Atoms (SIESTA 4.1.5) package [29]. This ab initio software solves Kohn–Sham equations determining its eigenfunctions as a linear combination of the numeric atomic orbitals, of a finite, predetermined size in the radial direction [30,31]. The angular dependence is represented by spherical harmonics, i.e., *s*, *p*, and *d* orbitals. The *s* and *p* orbitals of gallium and nitrogen atoms are represented by triple zeta functions. In the case of gallium, the internal *d* shell electrons are incorporated in the valence electron set, and their eigenstates are represented by single zeta functions. The size of the functional basis was reduced by the Troullier–Martins pseudopotentials replacing the electrons belonging to the atomic cores [32,33]. The integration over the k-space was replaced by the sum over the Monkhorst–Pack special points grid $\left(1\times 1\times 1\right)$ in the momentum space [34], which is essentially the solid-state application of Gauss quadratures [34]. The GGA-PBE (PBEJsJrLO) functional with the parameters β, µ, and κ fixed by the jellium surface (Js), jellium response (Jr), and Lieb–Oxford bound (LO) criteria, respectively, was used as described by [35,36,37]. An additional real-space grid was used for the fast calculation of the multicenter overlap integrals. The set density was controlled by the energy cutoff in the reciprocal space. The cutoff value in the reciprocal space corresponded to 410 Ry, which is approximately equivalent to a grid spacing in the real space of about 0.08 Å.

The electric potential, i.e., the Poisson equation was solved by the fast Fourier transform (FFT) series method. Therefore, periodic boundary conditions had to be imposed on the solutions. The interaction between slab copies was canceled by additional contribution to the potential offsetting the slab dipole [38,39].

SCF loop termination condition was that the level of the difference for any element of the density matrix was set not higher than 10^−4^. The atoms’ relaxation terminated when the forces acting on the atoms were not higher than 0.005 eV/Å. The ab initio simulated GaN lattice constants were a=3.21 Å and c=5.23 Å. These values are in good agreement with the experimental data for GaN: a = 3.189 Å, c = 5.186 Å [40]. The band correction scheme of Ferreira et al. known as GGA-1/2 was used, giving proper band gap energies, effective masses, and band structures [41,42]. The ab initio bandgap was $E_{g}^{DFT}\left(GaN\right)=3.47$ eV, which is in excellent agreement with the low-temperature experimental value, $E_{g}^{DFT}\left(GaN\right)=3.47$ eV [43,44]. The electronic properties were obtained in a modified Ferreira’s scheme for which positions of atoms and a periodic cell were first obtained using the PBEJsJrLO exchange-correlation functional.

## 3. The Results

Semiconductor surfaces undergo intensive reconstructions. A principal factor in the reconstruction is energy optimization. This is related to the fact that the direct termination surface entails the existence of quantum high-energy states related to the absence of an overlap with missing neighbors [1]. In principle, these states (called broken-bond states in the simplified description) can be emptied by the charge transfer to the bulk states. Inevitably, that leads to surface charge separation, which is energetically costly; thus, this route is not optimal. Typically, the excess charge at the surface is of order of 0.01 elementary charge for a single GaN(0001) surface atom [28]. This is much lower than the charge associated with a Ga broken bond, which is estimated to be $\left(1/4\right)e;$ therefore, the surface excess charge layer transfer is not expected to be an efficient route to surface energy minimization.

An alternative way to reduce the energy of surface broken-bond states is to reshuffle the topmost atoms that create a higher-state overlap, thus lowering their energy. The exemplary case is the low-temperature $\left(7\times 7\right)$ reconstruction of the silicon Si(111) surface that includes the creation of dimers in the top layer [2]. Thus, such a surface reconstruction depends critically on the atom bonding. The structure is the result of the energetic interplay between electronic and structural contributions. Generally, additional energy related to the strain is compensated by the electron energy decrease. As shown recently, gallium nitride is not a typical ${sp}^{3}$-bonded semiconductor—its valence band is composed of two separate subbands: higher, composed of ${Ga4s4p}^{3}-N2p$ states, and lower, composed of Ga3d−N4s states [45,46]. These states play a different role in the bonding. The lower-energy subband, composed of the collection of five Ga3d and one N4s orbitals, is isotropically cohesive. On the contrary, the higher-energy subband is composed of directional ${Ga4s4p}^{3}$ and off-directional N2p orbitatals. Thus, the latter’s contribution is highly directional and is solely responsible for the arrangement of Ga and N atoms into a tetrahedral wurtzite lattice. Hence, due to the presence of different bonding, it is relatively easy to switch from the *sp*^3^ to other configurations at the surface.

The obtained surface symmetry pattern could be affected by the size of the simulated system. Generally, it is expected that semiconductor surfaces are reconstructed in order to comply with the electron counting rule (ECR) [4,15]. The surface reconstruction and underlying ECR argument is a result of the energy optimization by the motion of the surface’s topmost atomic layers. The atoms are moving in such a way that the resulting change in the quantum states lowers the energy of the occupied states and increases the energy of the empty ones. The latter do not contribute to the total system energy, while the change in the occupied ones leads to a total energy decrease. The identification of the bonding within the topmost layer could be made employing partial density of state plots and crystal orbital Hamilton population (COHP) diagrams [47,48].

### 3.1. Stoichiometric GaN Bulk

**Figure 1:**
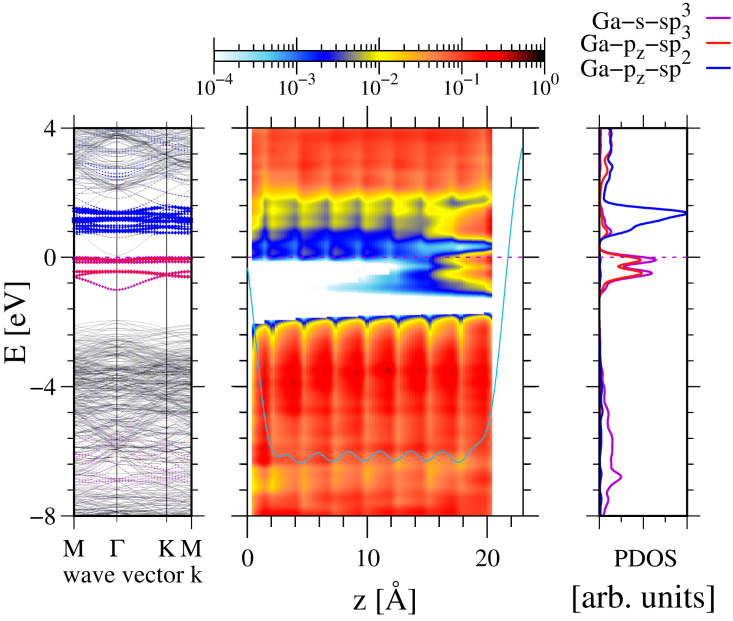
Electronic properties of $\left(4\times 4\right)$ 8 double Ga-N atomic layer (DAL) thick slab, representing stoichiometric Ga-terminated GaN(0001) surface: left—band diagram in momentum space, center—energy bands in real space, plotted along c-axis, right—partial density of states (PDOS) plotted for the top-layer Ga atoms. The blue and magenta represent surface layer Ga atoms: $Ga4{sp}^{3}$ hybridized and $Ga4p_{z}$ orbital states in conjunction with $Ga4{sp}^{2}$ hybridized states, respectively.

The structural and electronic properties of the flat GaN(0001) surface were investigated by ab initio simulations using several slab sizes: $\left(4\times 4\right)$, $\left(6\times 4\right)$, and $\left(8\times 4\right)$. The selection was partially determined by the earlier results indicating existence of a $\left(2\times 1\right)$ reconstruction [7,8]. The electronic properties obtained in a $\left(4\times 4\right)$ slab are presented in Figure 1 using the methods previously developed [11]. These data indicate that the surface state bands attributed to broken Ga bonds of GaN(0001) surface are different from the previously published ones [26,27,28]. The presently obtained surface states actually belong to two bands, separated by a gap of 0.3 eV. The lower surface state band is fully occupied, and the upper is empty; thus, the Fermi level is located at the top of the lower band, i.e., effectively in the gap. The upper surface band (empty) is identified as belonging to the surface gallium atoms’ $Gap_{z}$ orbitals. The lower surface band (occupied) is composed of the subbands that are associated with the top-layer Ga atoms having $Ga{sp}^{3}$ (upper subband) and $Ga{sp}^{2}$ (lower subband) hybridized orbitals. These subbands are not separated by the energy gap; they overlap in the energy scale. In fact, the energy difference in their clearly distinguishable density of states (DOS) maxima is about 0.3 eV. Both subbands are occupied; thus, such a stoichiometric GaN(0001) surface is semiconducting.

**Figure 2:**
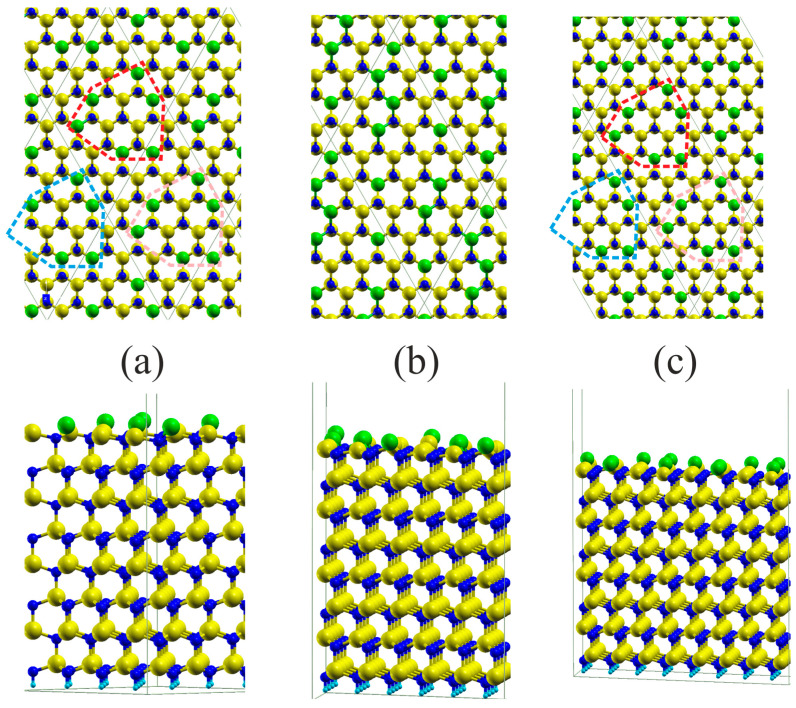
Gallium nitride 8 Ga-N double atomic layer (DAL) thick slabs representing Ga-terminated GaN(0001) surface: (**a**) $\left(4\times 4\right)$ slab, (**b**) $\left(6\times 4\right)$ slab, (**c**) $\left(8\times 4\right)$ slab. The upper and lower rows present the top and side view, respectively. The balls represent the following atoms: blue—nitrogen, yellow—gallium, green—surface layer gallium $\left({sp}^{3}\right)$ hybridized, cyan—hydrogen termination pseudoatoms. The red broken lines mark the basic units of $\left(4\times 4\right)$ reconstruction pattern.

As shown in Figure 2, these electronic properties are closely related to the structural properties of the surface. The gallium topmost surface layer atoms are divided into two sets: the first—located approximately in standard lattice sites and the second—located in the plane of the topmost nitrogen atoms. Therefore, the first set is identified as associated with the ${sp}^{3}$ hybridization and the second with ${sp}^{2}$ hybridization. In addition, the $Ga{4p}_{z}$ state in the second set is higher in energy, located above Fermi level, and therefore it is not occupied as shown in Figure 1. The occupied states are different in energy, those of ${sp}^{2}$ hybridization have more contribution from the *Ga-s* state; therefore, they have lower energy. Those of ${sp}^{3}$ hybridization have slightly higher energy; thus, we observe two peaks in the PDOS plot.

As shown in Figure 2a, in the case of a $\left(4\times 4\right)$ slab, the set of ten $\left({sp}^{3}\right)$ hybridized Ga atoms is approximately located at the standard GaN lattice sites, while the remaining six $\left({sp}^{2}\right)$ hybridized Ga atoms are positioned in the topmost nitrogen atom plane. This could be understood from ECR analysis as follows: denote the fraction of surface Ga atoms of ${sp}^{2}$ hybridization by x; therefore, the fraction of ${sp}^{3}$ hybridization Ga surface atoms is $\left(1-x\right)$. The latter atoms have two broken-bond ${sp}^{3}$ states that are occupied by the electrons; the former have two broken-bond $p_{z}$ states that are empty (${sp}^{2}$ states are occupied). All the Ga-N bonds are occupied; therefore, the excess charge for the surface Ga top-layer atom broken bond (i.e., vertical) is $\left(3/4\right)$ for a single atom. The ECR-based electron charge balance thus assumes occupation by two electrons in the vertical bond state in the ${sp}^{3}$ configuration, i.e., a fraction of (1 − *x*) sites and 0 electrons in $p_{z}$ orbital in ${sp}^{2}$ hybridized atom, i.e., a fraction of *x* sites:

(1) $$\left(1-x\right)\times 2+0\times x=\frac{3}{4}$$

From this relation, it follows that the fraction of ${sp}^{2}$ hybridized Ga atoms is $x=\frac{5}{8}$. The remaining fraction $1-x=\frac{3}{8}$ Ga atoms is ${sp}^{3}$ hybridized. Accordingly, in the $\left(4\times 4\right)$ slab, 10 Ga atoms are located in the N atom plane, while the remaining 6 Ga atoms are predicted to be located in higher, lattice-compatible positions. The distribution of Ga atoms in the topmost plane remains in full agreement with the ECR prediction. Similarly, in the case of a $\left(6\times 4\right)$ slab, 15 and 9 Ga atoms are in the predicted position in full agreement with the ECR argument. And, finally, in the case of an $\left(8\times 4\right)$ slab, 20 and 12 Ga atoms are in ${sp}^{2}$ and ${sp}^{3}$ hybridization, respectively. In summary, the ab initio results fully confirm the prediction based on the ECR analysis; therefore, the atoms are hybridized due to the electron redistribution.

As shown in Figure 1, in the case of $\left(4\times 4\right)$ and $\left(8\times 4\right)$ slabs, the emerging $\left(4\times 4\right)$ reconstruction is compatible with the slab periodicity. In the case of a $\left(6\times 4\right)$ it is not; therefore, this reconstruction is not observed. Thus, successful simulations of the surface reconstruction require proper choice of the simulation slab. Therefore, for a large-size surface $\left(4\times 4\right),$ the reconstruction based on electron redistribution is an energetically stable configuration of the GaN(0001) stoichiometric surface.

### 3.2. GaN Slab with n-Type and p-Type Doping

An important issue to be solved is related to the charge transfer between the surface sites and also charge exchange between the surface and the bulk. This was investigated by simulations of p-type and n-type doped slabs. The p-type doping was simulated by te introduction of the two Mg substitutional atoms in Ga sites, which leads to the total deficit of the two electrons in the valence band, which, in principle, should be sufficient to evacuate two electrons from the ${sp}^{3}$ hybridized Ga atoms and change the reconstruction of the surface. Similarly, substitution of Ga by Si atoms leads to the surplus of the two electrons in valence charge so that these additional charges should be located on the Ga surface bonds, thus increasing number of ${sp}^{3}$ hybridized Ga atoms by unity. This could affect also the potential distribution within the slab.

**Figure 3:**
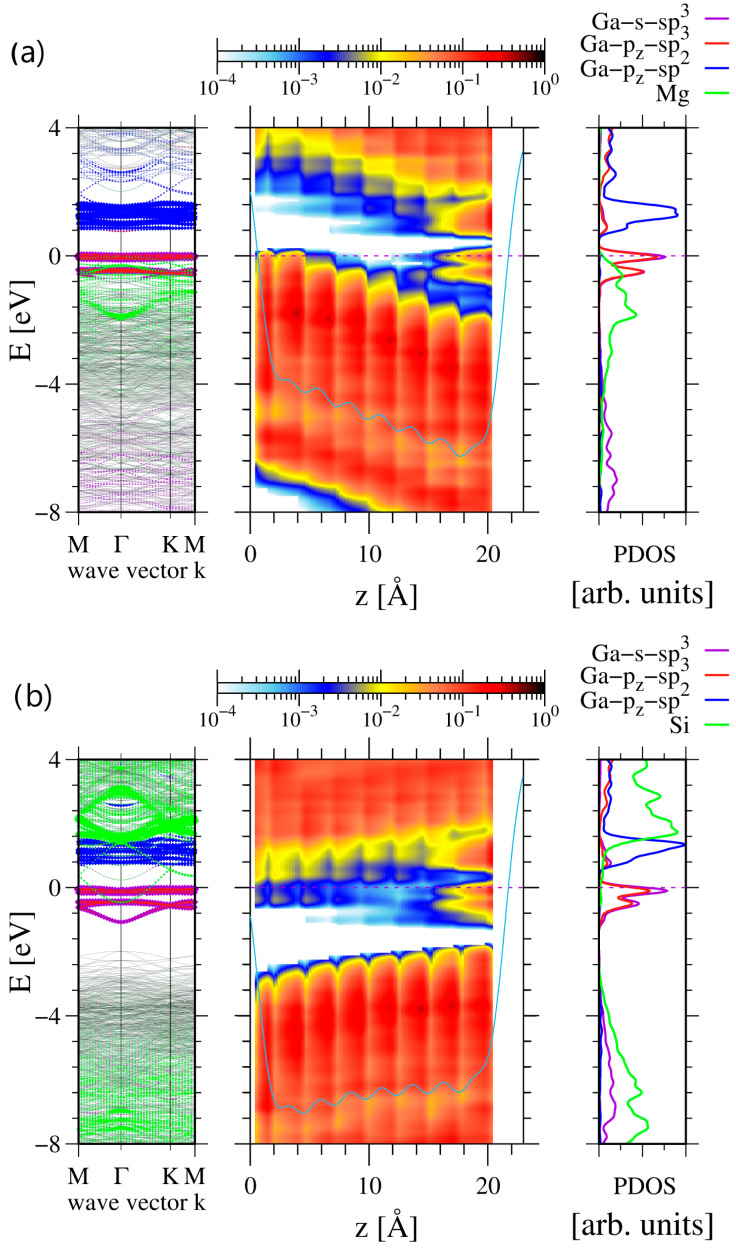
Electronic properties of $\left(4\times 4\right)$ 8 double Ga-N atomic layer (DAL) thick slab, representing Ga-terminated GaN(0001) surface: (**a**) doped by Ga substitution by two Mg atoms (0.0156 at%), (**b**) by Ga substitution by two Si atoms (0.0156 at%). The notation is as in Figure 1. Additional green lines represent Mg and Si states, multiplied by factor of 10 in order to visualize their presence.

As shown in Figure 3, the electric properties of the GaN(0001) surface slabs are considerably affected by the doping, inducing a considerable field along the c-axis. In the case of Mg doping, the electric field estimated from the slope of the potential is $\vec{E}\left(Mg\right)\;≅6\times 10^{-2}\;V/Å=6\;\mathrm{MV}/\mathrm{cm}$. This field emerges due to the positive charge accumulated at the surface, which can be assessed from Gauss’ law $q_{Mg}=\varepsilon_{o}\epsilon_{GaN}\;\left|\vec{E}\left(Mg\right)\right|\;S$. The $\left(4\times 4\right)$ slab surface area is $S=16\;a_{GaN}^{2}\sqrt{3}/2=1.406\times 10^{2}{Å}^{2}=1.406\times 10^{-19}\;m^{2}$ in which the ab initio lattice constant was used, i.e., a=3.194 Å. The other values used were vacuum permittivity $\varepsilon_{o}=8.854\times 10^{-12}\;F/m$ and GaN dielectric permittivity $\epsilon_{GaN}=10.28$. The total charge was, therefore, $q_{Mg}=7.68\times 10^{-20}\;C=0.48\;e$, i.e., much below the charge 2 e necessary for the full occupation of two ${sp}^{3}$ hybridized states necessary for the transformation of a single Ga surface atom. As shown by the band diagram, the positive charge at the surface is smaller because the remaining positive charge is located in the empty valence band states, which are located in the left part of the slab presented in Figure 3a. This is further confirmed by the different occupation of the higher peak related ${sp}^{3}$ subband in Figure 3a,b. In the case of Mg doping (Figure 3a), the higher peak is approximately half occupied, while, in the case of Si doping (Figure 3b), this occupation is close to complete. Therefore, the fractional shift from ${sp}^{2}$ to ${sp}^{3}$ hybridized configuration is observed.

Similarly, the accumulation of the negative surface charge originated from the introduction of the two Si substitutional atoms may be assessed. The field related to Si doping is not uniform within the slab, indicating an important role of the bulk charge. The field at the surface is much lower $\vec{E}\left(Si\right)\;≅2\times 10^{-2}\;V/Å=2\;\mathrm{MV}/\mathrm{cm}$; therefore, the total charge at the slab surface is $q_{Si}=2.56\times 10^{-20}\;C=0.16\;e,$ which is again not sufficient to induce a change in the surface symmetry.

**Figure 4:**
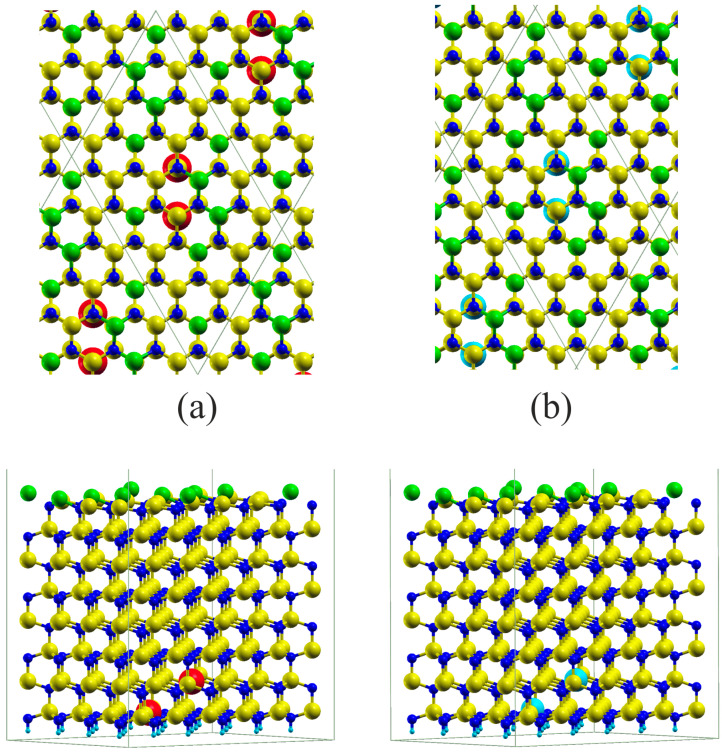
Gallium nitride $\left(6\times 4\right)$ 8 DAL thick slabs representing Ga-terminated GaN(0001) surface doped with the pair of Si substitutional atoms. Most symbols are as in Figure 1. Additionally, red and cyan balls represent Mg and Si atoms, (**a**) and (**b**) subfigure respectively.

**Figure 5:**
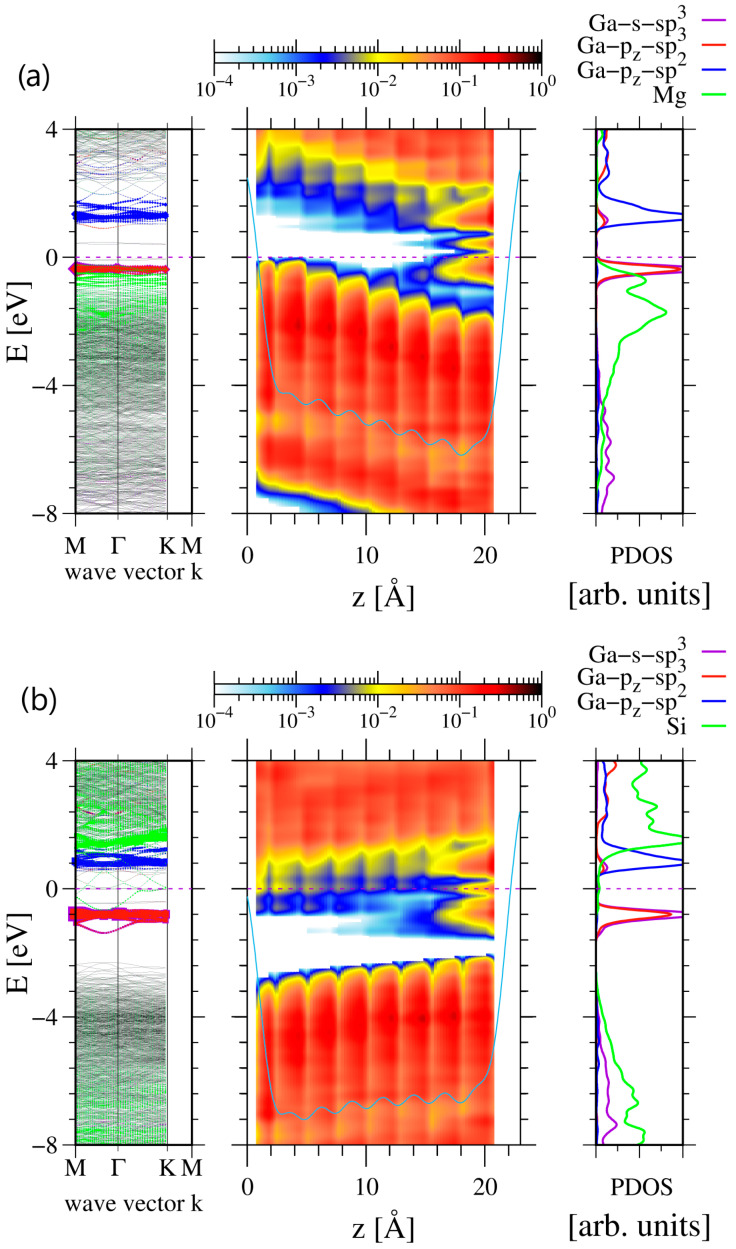
Electronic properties of $\left(6\times 4\right)$ 8 double Ga-N atomic layer (DAL) thick slab, representing Ga-terminated GaN(0001) surface: (**a**) doped by Ga substitution by two Mg atoms (0.0104 at%), (**b**) by Ga substitution by two Si atoms (0.0104 at%). The notation is as in Figure 1. Additional green lines represent Mg and Si states, multiplied by factor of 10 in order to visualize their presence.

An additional investigation of the doping of a $\left(6\times 4\right)$ slab to p-type and n-type was made by substituting the pair of Ga atoms by Mg and Si, respectively. The results, plotted in Figure 4, indicate that, in the case of n-type doping, the number of surface layer Ga atoms in ${sp}^{3}$ hybridization was increased to 10, thus finally confirming the decisive role of the charge in GaN(0001) reconstruction. This could be confirmed by the plots of the band diagrams in Figure 5.

In the case of Mg doping, the field is $\vec{E}\left(Mg\right)≅5\times 10^{-2}V/Å=5\;\mathrm{MV}/\mathrm{cm}$, the surface is $S=24a_{GaN}^{2}\sqrt{3}/2=2.109\times 10^{2}{Å}^{2}=2.109\times 10^{-19}\;m^{2},$ which gives the total charge $q_{Mg}=1.15\times 10^{-19}C=0.72e$, i.e., still below the critical charge of 2e; thus, only the fractional change is observed. In the case of Si, the numbers are $\vec{E}\left(Si\right)≅2\times 10^{-2}V/Å=2\;\mathrm{MV}/\mathrm{cm}$, which gives the total charge as $q_{Si}=3.84\times 10^{-20}C=0.24e$. Nevertheless, as shown in Figure 4, the number of ${sp}^{3}$ hybridized atoms increases to 10.

In general, the symmetry of the surface reconstruction results from the charge and strain energy optimization. In the case of the slab and reconstruction compatibility, the system is more stable; therefore, the change of the reconstruction is more difficult to induce by doping. In the case of an incompatible $\left(6\times 4\right)$ slab, the number of the differently hybridized atoms could be changed more easily.

The driving force of the above reconstruction is the difference in the energy of the Ga−s and Ga−p orbitals. Therefore, this reconstruction can occur for the surfaces of typical III-V semiconductors such as GaAs(111). In the case of the nitrides, Ga-terminated surfaces can undergo this reconstruction such as the investigated GaN(0001). On the contrary, nitrogen-terminated surfaces will not undergo this reconstruction in the N-terminated $GaN\left(000\bar{1}\right)$ surface [26,46]. Despite large difference in the energies of the N−s and N−p orbitals, the basic bonding of the solid does not involve ${sp}^{3}$ hybridization of nitrogen; therefore, such reconstruction is not observed.

## 4. Summary and Conclusions

The best way to summarize the results obtained in this work is to use a scheme presenting: (i) the state of art before the publication, (ii) the results of the present work, and (iii) the state of art after publication.

The state of the art, related to this paper’s subject, before its publication may be summarized as follows:

(a) Reconstruction of the semiconductor surface is the result of energy optimization by the saturation of broken bonds.
(b) Reconstruction can be removed only by adsorption of the species saturating the broken bonds locally.

The results obtained in this paper may be summarized as follows:

(a) Charge balance controls the mixed reconstruction in which, for the stoichiometric case, (3/8) surface Ga atoms remain in the standard positions with ${sp}^{3}$ hybridized bonding, while the other (5/8) surface Ga atoms are located in plane of N atoms with ${sp}^{2}$ hybridized bonding.
(b) Charge balance controls the energy gain in the reconstruction of the GaN(0001) surface by the selection of the maximal fraction the ${sp}^{2}$ hybridized orbitals that leaves the associated $p_{z}$ orbitals empty. The remaining ${sp}^{3}$ hybridized orbitals have higher energy; thus, they do not participate in energy optimization.
(c) Reconstruction induced strain optimization in conjunction with charge balance leads to $\left(4\times 4\right)$ symmetry of the stoichiometric GaN(0001) surface.
(d) Doping in the GaN bulk affects or even destroys surface reconstruction symmetry by a mere change in the charge balance.

The state of art after publication can be described as follows:

(a) The charge transfer energy reduction mechanism by transition to occupation of ${sp}^{2}$ bonding is universal, typical for all surfaces terminated by ${sp}^{3}$ bonded atoms.
(b) Reconstruction depends on the charge balance, in the surface and in the bulk; thus, it is a global effect.
(c) Adsorption may affect reconstruction globally by affecting the charge balance even at locations far from the adsorption site.

In conclusion, it is stated that these results prove a new control by charge balance of the energy optimization of semiconductor surfaces via reconstruction. This mechanism was proved in case of simple ${sp}^{3}$ to ${sp}^{2}$ bonding transition. More elaborate bonding, such as bridge formation, needs elucidation by the investigation of the charge contribution to the energy optimization in the future.

## Data Availability

The data will be available on request from corresponding author.
